# C/EBPα deficiency in podocytes aggravates podocyte senescence and kidney injury in aging mice

**DOI:** 10.1038/s41419-019-1933-2

**Published:** 2019-09-17

**Authors:** Liwen Zhang, Fangfang Zhou, Xialian Yu, Yufei Zhu, Ying Zhou, Jian Liu, Yunzi Liu, Qingyang Ma, Yuchao Zhang, Weiming Wang, Nan Chen

**Affiliations:** 1 Department of Nephrology, Ruijin Hospital, Shanghai Jiao Tong University School of Medicine, 200025 Shanghai, P.R. China; 2 Institute of Nephrology, Shanghai Jiao Tong University School of Medicine, 200025 Shanghai, P.R. China; 3 The Key Laboratory of Stem Cell Biology, Shanghai Institutes for Biological Sciences, Chinese Academy of Sciences, 200031 Shanghai, P.R. China

**Keywords:** Senescence, Preclinical research

## Abstract

Kidney aging leads to an increased incidence of end-stage renal disease (ESRD) in the elderly, and aging is a complex biological process controlled by signaling pathways and transcription factors. Podocyte senescence plays a central role in injury resulting from kidney aging. Here, we demonstrated the critical role of C/EBPα in podocyte senescence and kidney aging by generating a genetically modified mouse model of chronological aging in which *C/EBPα* was selectively deleted in podocytes and by overexpressing C/EBPα in cultured podocytes, in which premature senescence was induced by treatment with adriamycin. Moreover, we illuminated the mechanisms by which podocyte senescence causes tubular impairment by stimulating HK-2 cells with bovine serum albumin (BSA) and chloroquine. Our findings suggest that *C/EBPα* knockout in podocytes aggravates podocyte senescence through the AMPK/mTOR pathway, leading to glomerulosclerosis, and that subsequent albuminuria exacerbates the epithelial–mesenchymal transdifferentiation of senescent tubular cells by suppressing autophagy. These observations highlight the importance of C/EBPα as a new potential target in kidney aging.

## Introduction

As the global population has aged, the incidence of kidney disease in the expanding geriatric population has grown proportionally. A progressive decline in the physiological function of all organs and systems is a common characteristic of aging^1^. In the kidneys, this decline appears to involve an ~10% decrease in the glomerular filtration rate per decade after the age of 35^2^. Histologically, this change manifests as a loss of functional nephrons, including podocyte depletion, glomerulosclerosis, tubular atrophy, microvascular rarefaction, and interstitial fibrosis, as well as a compensation of the remaining nephrons. Cytologically, aging induces the accumulation of damaged organelles, mitochondria, and protein aggregates within renal cells, causing senescence and dysfunction. Extending the lifespan has been a mysterious and attractive topic throughout human history.

CAAT enhancer-binding proteins (C/EBPs) are a family of transcription factors belonging to the basic region leucine zipper (bZIP) family of proteins. They are widely expressed in liver, fat, and hematopoietic cells and other terminally differentiated cells^3^. Previous studies have found that C/EBPα is broadly expressed in all cells in the glomeruli and is especially highly expressed in podocytes^4^. C/EBPα controls a wide range of cellular processes, including cellular proliferation and differentiation, energy metabolism, inflammation, and autoimmunity^5^. In particular, our previous study has shown that C/EBPα maintains podocyte integrity in an experimental FSGS model in vivo and in vitro^4^. However, the effects of C/EBPα in podocyte senescence and kidney aging remain unknown.

Here, we demonstrated the protective role of C/EBPα in podocyte senescence and kidney aging by generating a genetically modified mouse model of chronologic aging in which *C/EBPα* was selectively deleted in podocytes and by overexpressing C/EBPα in cultured podocytes and inducing senescence by adriamycin. Our findings suggest that *C/EBPα* knockout in podocytes aggravates podocyte senescence, which exacerbates further glomerulosclerosis and tubular injury in aging mice. These observations highlight the importance of C/EBPα as a new potential target in renal aging.

## Materials and methods

### Animal experiments

Animal maintenance and experimental procedures were approved by the Animal Care Committee of Ruijin Hospital, Shanghai Jiao Tong University School of Medicine (Shanghai, China). Mice were housed in a specific pathogen-free room at a constant temperature of 22 ± 2 °C and a constant humidity of 50 ± 5% under a 12-h day/night cycle. *Podocin-Cre* and *floxed Cebpa* (*Cebpa*^*flox/flox*^) transgenic mice on the C57BL/6 background were crossed to generate *Cebpa*^*flox/flox*^*; podocin-Cre* mice (hereafter referred to as *Cebpa*^*Pod-f/f*^ mice), and they were bred and genotyped in our laboratory as described previously^4^. For studies involving the deletion of *C/EBPα* in podocytes in aging mice, mice were divided into the following four groups: *Cebpa*^*Pod-+/+*^ mice that were killed at 12 weeks and 20 months of age (the WT-Young group and the WT-Aging group, respectively) and *Cebpa*^*Pod-f/f*^ littermates (the KO-Young group and the KO-Aging group). Mice were given free access to chow and water.

### Cell culture

HK-2 cells were obtained from American Type Culture Collection (Manassas, VA, USA) and cultured in DMEM/F12 medium with 10% fetal bovine serum. Immortalized mouse podocytes were kindly provided by Professor John Cijiang He (Department of Nephrology, Icahn School of Medicine at Mount Sinai, New York, NY, USA), cultured as previously described^6^, and differentiated at 37 °C for 3 days. Podocytes were transfected as previously described^6^. *C/EBPα* NGFR overexpression plasmid and its negative control were gifts from Ellen Rothenberg (Addgene plasmid #44627, Watertown, MA, USA)^7^.

### Metabolic and physiologic parameters

Before the mice were euthanized, they were provided water ad libitum, and 24-h urine was collected in metabolic cages. The urinary albumin concentration was measured by using a Mouse Albumin ELISA Quantitation Set (Bethyl Laboratories, Inc., Montgomery, TX, USA). The urinary creatinine concentration in the same sample was measured by using the QuantiChrom^TM^ Creatinine Assay Kit (BioAssay Systems, Hayward, CA, USA) according to the manufacturer’s protocol.

### Kidney histopathology

The kidneys were removed from anesthetized mice and were immediately fixed in 4% paraformaldehyde, embedded in paraffin, and sectioned at 4 μm. The sections were stained with periodic acid-Schiff (PAS) and Trichrome Masson. PAS micrographs were observed to estimate the glomerular tuft and mesangial areas. The cross-sectional area of the glomerular tuft was determined from outlines of the tuft using the program Adobe Photoshop 7.0 (Adobe Systems, Inc., San Jose, CA). The mesangial fraction was calculated as the ratio of the mesangial area to the area of the glomerular tuft^4^. Histopathological characteristics were quantified in a blinded fashion based on at least ten glomeruli per mouse at a magnification of ~×400 (DM1000, Leica, Germany).

### Transmission electron microscopy

Renal cortical tissues were fixed in 2% glutaraldehyde in phosphate-buffered solution (pH 7.4). Samples were further incubated with 2% osmium tetroxide in phosphate-buffered solution (pH 7.4) for 2 h at 4 °C. Ultrathin sections were stained with lead citrate and uranyl acetate and viewed on a HT770 transmission electron microscope (Hitachi, Japan) at an accelerating voltage of 80 kV. ImageJ 1.51k software (National Institutes of Health, rsb.info.nih.gov) was used to measure the glomerular membrane thickness. After separating out the various segments and leaving only the GBM, we used BoneJ, an ImageJ plugin for bone image analysis, to measure the GBM thickness as previously described^8^.

### Total RNA extraction and quantitative real-time PCR

The total RNA from renal cortical tissues was extracted by using TRIzol (Applied Biosystems, Waltham, MA, USA). The RNA concentration was measured by an ND-1000 spectrophotometer (NanoDrop Technologies, Wilmington, DE, USA). First-strand cDNA synthesis was performed by using 2 μg of RNA and the High-Capacity cDNA Reverse Transcription Kit (Applied Biosystems) according to the manufacturer’s instructions. Real-time quantitative RT-PCR was performed using SYBR^®^ Premix Ex Taq™ (TAKARA, Japan) and the StepOnePlus real-time PCR system (Applied Biosystems). The sequences of the mouse primers for *vimentin*, *β-actin*, *p21*^*CIP1*^*, p27*^*KIP1*^, and *p15*^*INK4b*^ are available on request. The sequences of the oligonucleotide primers for *nephrin*, *synaptopodin*, *podocin*, *WT-1*, and *podocalyxin* were also available as previously described^4^. The expression levels of each mRNA were calculated after normalizing to those of *β-actin*. The results were analyzed by using the comparative cycle threshold (2^–∆∆Ct^) method.

### Immunohistochemistry staining

IHC staining was performed on paraffin-embedded kidney sections following standard procedures by incubating the sections in a primary antibody against LC3B (Cell Signaling Technology, Inc., Danvers, MA, USA), E-cadherin (Abcam, UK), α-SMA (Santa Cruz Biotechnology, CA, USA) and FSP1 (S100A4, Abcam) at 4 °C overnight. After washing, the sections were incubated with biotinylated secondary antibodies, followed by incubation with an avidin–biotin–peroxidase complex for DAB substrate development using the ABC kit (Vector Laboratories, Burlingame, CA, USA) at room temperature, and they were mounted using Aqua PolyMount (Polysciences, Inc., Warrington, PA, USA). Images were acquired by using a Leica DM1000 microscope with a digital camera.

### Immunofluorescence

After antigen retrieval and blocking, 5-mm kidney paraffin sections were incubated overnight at 4 °C with goat anti-synaptopodin (Santa Cruz Biotechnology), mouse anti-WT-1 (Santa Cruz Biotechnology), mouse anti-p16^*INK4a*^ (Abcam), or mouse anti-C/EBPα primary antibodies (Santa Cruz Biotechnology). After washing, the sections were incubated with FITC-conjugated donkey anti-mouse/anti-goat secondary antibodies. The sections were examined using an AxioVert A1 microscope (Zeiss, Germany) with a digital camera. ImageJ 1.51k software (National Institutes of Health) was used to measure the level of immunostaining in the glomeruli. First, the images were converted into 8-bit grayscale images. The glomerular regions were selected to measure the area. After grayscale inversion, the optical density (OD) was calibrated. Then, the positively stained regions in the glomeruli were selected with appropriate settings and concordant thresholds, the cells were counted, the positively stained area was calculated, and both measurements were normalized to the glomerular areas.

### Senescence-associated β-galactosidase staining

Senescence-associated β-galactosidase (SA-β-gal) staining of renal tissue was performed in the OCT freezing medium and SA-β-gal staining of podocytes was performed in glass dishes (Thermo Scientific, USA) using the Senescence β-Galactosidase Staining Kit (cat. no. 9860, Cell Signaling Technology, USA). Briefly, 10-µm tissue cryosections were fixed at room temperature for 5 min in fixative solution and then processed according to the manufacturer’s protocol. Images were acquired using a Leica DM1000 microscope and quantified using ImageJ 1.51k software.

### Western blot analysis

Renal cortical tissues were ground and lysed, and cells were collected and lysed in RIPA buffer containing the protease inhibitor cocktail. Equal amounts of protein samples were loaded on SDS polyacrylamide gels, transferred to PVDF membranes (Millipore, MA, USA), probed with antibodies, and visualized with the Luminescent Imaging Workstation (Tanon, China). The band intensities were quantified using ImageJ. The following antibodies were used: rabbit anti-nephrin antibody (Abcam), mouse anti-E-cadherin antibody (Abcam), mouse anti-α-SMA antibody (Santa Cruz Biotechnology), rabbit anti-p62 antibody (Cell Signaling Technology, Inc.), and rabbit anti-LC3B antibody (Cell Signaling Technology, Inc.). Mouse anti-β-actin antibody (Sigma, MA, USA) or mouse anti-GAPDH antibody (Abcam) was used as loading controls.

### Statistical analyses

The group data are expressed as the mean ± standard error of the mean (SEM). Comparisons between two groups were performed using an unpaired *t* test after determining the distribution and variance of the data. One-way analysis of variance (ANOVA) followed by Tukey’s multiple-comparison test was used when more than two groups were present. All tests were two-tailed, and *P* < 0.05 was considered to be a statistically significant result.

## Results

### Characterization of C/EBPα expression in a podocyte-specific *C/EBPα*-null mouse model and in a chronologic aging mouse model

**Fig. 1:**
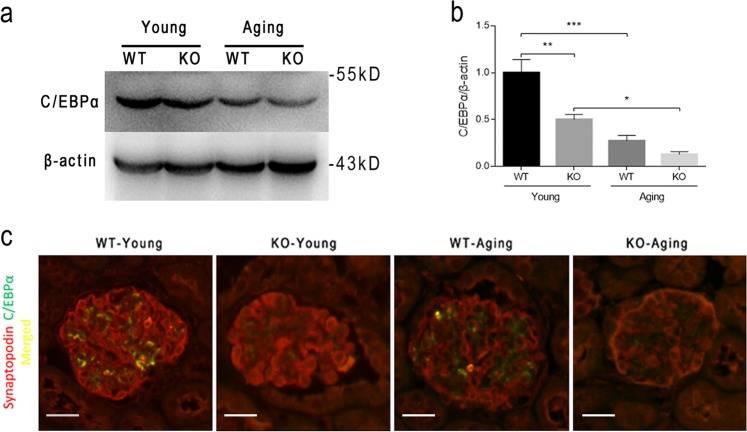
Podocyte-specific *C/EBPα* knockout in aging mice. **a** Western blot analysis was used to assess an altered expression in renal cortical tissues from aging mice and to confirm the disruption of C/EBPα expression in *C/EBPα*^*Pod-f/f*^ mice. β-actin was used as a loading control. **b** The quantification of the average band intensity for C/EBPα. The values of expression in the *C/EBPα*^*Pod-+/+*^ young (WT-Young) group were set as 1. The results are presented as the means ± SEMs. One-way analysis of variance (ANOVA) followed by Tukey’s multiple-comparison test was used. *P < 0.05, **P < 0.01, and ***P < 0.001 between the indicated groups. **c** Representative immunostaining micrographs of C/EBPα (green) and synaptopodin (red, indicating podocytes) show decreased C/EBPα expression in podocytes upon aging and the deletion of C/EBPα upon knockout. The scale bars = 20 μm. All images are merged projections of 594- and 488-nm Z-serial channels

We crossed *Cebpa-floxed* (*Cebpa*^*flox/flox*^) mice with transgenic mice expressing podocyte-specific *Cre* recombinase under the *podocin* promoter (*podocin-Cre*) to generate podocyte-specific *C/EBPα* knockout (*Cebpa*^*Pod-f/f*^) mice and control (*Cebpa*^*Pod-+/+*^) mice as described previously^4^. There were 6–9 mice in each group. Our previous work has shown that under basal conditions, young mice with a podocyte-specific deletion of *C/EBPα* (KO-Young) are phenotypically normal up to the age of 12 months and born in accordance with the Mendelian law in a proportional male-to-female ratio^4^. Therefore, the young and aging groups were sacrificed at 3 and 20 months of age, respectively. To assess the change in C/EBPα expression with aging, we isolated the renal cortices for western blotting and found that the C/EBPα level in KO mice was robustly downregulated compared with that in WT mice, and aging also significantly reduced the C/EBPα levels (Fig. 1a, b). By examining the colocalization of C/EBPα and the podocyte marker synaptopodin by immunofluorescence, we found that C/EBPα in the kidneys was mainly expressed in podocytes and that *C/EBPα* expression in podocytes was specifically deleted in KO mice and downregulated upon aging (Fig. 1c).

### Targeted deletion of *C/EBPα* in podocytes accelerates podocyte senescence in chronologic aging mice

**Fig. 2:**
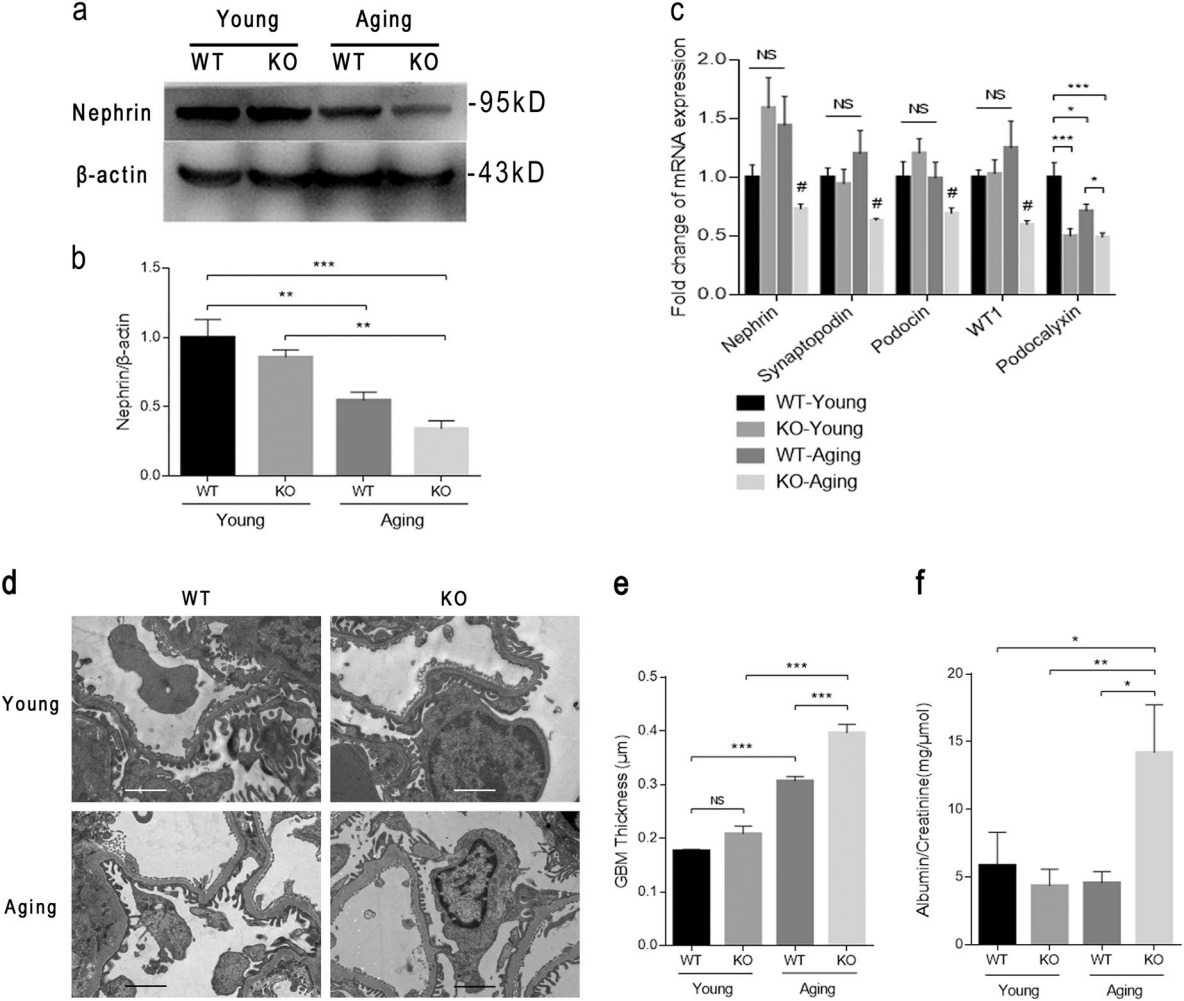
Conditional deletion of *C/EBPα* in podocytes exacerbates structural and functional impairment of senescent podocytes. **a** The expression of the podocyte marker nephrin in the renal cortices of young and aging wild-type and knockout mice (the WT-Young, KO-Young, WT-Aging, and KO-Aging groups) was determined by western blot analysis. **b** Densitometric analyses of the western blots in (**a**). The relative intensities of the bands were normalized to the intensities of the respective β-actin signal, and the value of the WT-Young group was set to 1. **c** The quantification of the mRNA expression levels of podocyte markers in the four groups described in (**a**). **d** Representative TEM micrographs of foot processes and the GBM in the WT-Young, KO-Young, WT-Aging, and KO-Aging groups. The scale bars = 2.0 μm. **e** The quantification of GBM thickness. **f** The development of proteinuria in aging *C/EBPα*^*Pod-f/f*^ mice (the KO-Aging group) was determined by the urinary albumin-to-creatinine ratio. The results are presented as the means ± SEMs. One-way analysis of variance (ANOVA) followed by Tukey’s multiple-comparison test was used. ^#^*P* < 0.05 compared with all the other groups, *P < 0.05, **P < 0.01, and ***P < 0.001, NS no significance between the indicated groups

We then investigated the structural and functional impairment of podocytes in aging mice. The western blot results in the renal cortices showed that the nephrin expression level was significantly downregulated upon aging and was decreased more markedly in the KO-Aging group than in the WT-Aging group (Fig. 2a, b). The mRNA levels of several podocyte markers, including *nephrin*, *synaptopodin*, *podocin*, *WT-1*, and *podocalyxin* in the isolated renal cortical tissues were next determined by real-time quantitative PCR analysis and were also reduced significantly in KO-Aging mice compared with WT-Aging mice (Fig. 2c). Moreover, transmission electron microscopy (TEM) micrographs also revealed podocyte foot process effacement and glomerular basement membrane (GBM) thickening upon aging and these morphological injuries were more serious in KO-Aging mice than in WT-Aging mice (Fig. 2d, e). In addition to aggravating morphological injuries, C/EBPα loss in podocytes also accelerated functional destruction induced by aging, which was demonstrated by the significantly increased urinary albumin-to-creatinine ratio in WT-Aging mice compared with that in the other three groups (Fig. 2f).

**Fig. 3:**
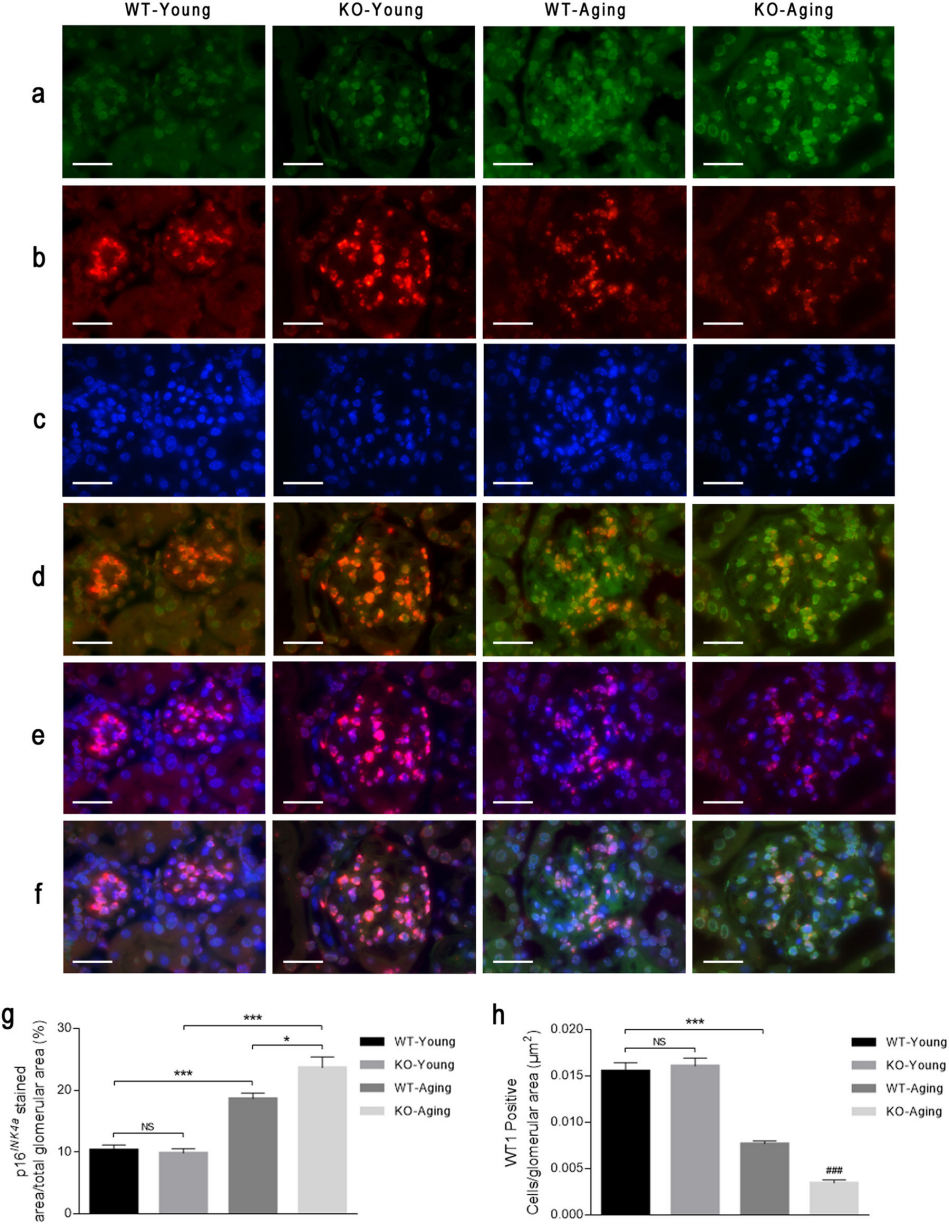
Conditional deletion of *C/EBPα* in podocytes enhances p16^*INK4a*^ accumulation and podocyte loss upon aging. Representative immunofluorescence micrographs of kidney sections from wild-type and C/EBPα knockout mice at 12 weeks and 20 months of age stained with primary antibodies against p16^*INK4a*^ (green) (**a**) and WT-1 (red) (**b**). The nuclei were counterstained with DAPI (blue). **c**, **d** The images are merged projections of 594- and 488-nm Z-serial channels. **e** The images are merged projections of 454- and 488-nm Z-serial channels. **f** The images are a merged image of the three channels. **g** The quantification of the p16^*INK4a*^ level in the glomeruli, and **h** the number of WT-1-positive cells per μm^2^ in the glomerular tuft area were determined by counting ten random glomeruli in each group from three independent experiments. The data are shown as the mean ± SEM. Statistical analysis was performed by one-way ANOVA followed by Tukey’s multiple-comparison test. *P < 0.05 and ***P < 0.001 between the indicated groups; ^###^*P* < 0.001 compared with the other three groups. In panels (**a**–**f**), the scale bars = 20 μm

To better understand whether an exacerbated podocyte impairment in KO-Aging mice is due to the acceleration of podocyte senescence by *C/EBPα* knockout, we first evaluated double immunostaining of the podocyte nuclear marker WT-1 and the cellular senescence marker p16^*INK4a*^. The micrographs show significantly increased p16^*INK4a*^ accumulation (Fig. 3a, g) and a reduced number of WT-1-positive cells (Fig. 3b, e, h) in the glomeruli upon aging, and *C/EBPα* knockout markedly enhanced these trends. The micrographs showing the colocalization of p16^*INK4a*^ and WT-1 indicated increased p16^*INK4a*^ accumulation in WT-1-positive cells in both WT and KO mice in the chronologic aging mouse model (Fig. 3d). Moreover, p16^*INK4a*^ accumulation in WT-1-positive cells was more serious in KO-Aging glomeruli than in WT-Aging glomeruli (Fig. 3d), although the percentage of WT-1-positive nuclei was reduced sharply in KO-Aging glomeruli (Fig. 3b, e, h).

**Fig. 4:**
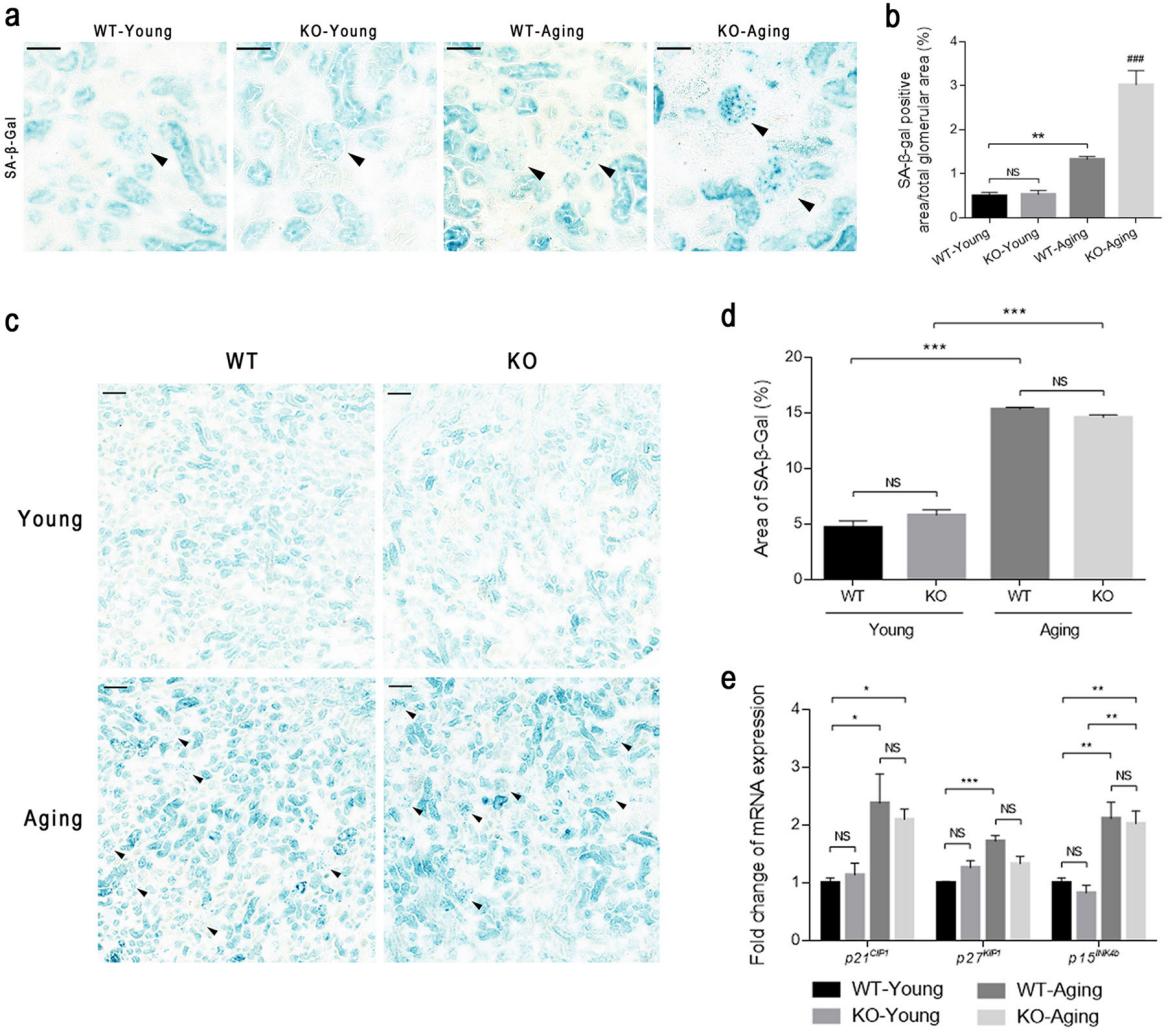
Senescence markers in the aging kidney. **a** Representative micrographs showing SA-β-gal staining in the glomeruli from wild-type and C/EBPα knockout mice at 12 weeks and 20 months of age. The triangles indicate the glomeruli. The scale bars = 50 μm. **b** The quantification of the SA-β-gal-positive area in the glomeruli was determined by counting ten random glomeruli in each group from three independent experiments. **c** Representative micrographs of SA-β-gal staining in the kidney cortices from the mice described in (**a**). The scale bars = 100 μm. **d** The SA-β-gal-positive area as a percentage of the renal cortical area in (**c**). Two random fields of view with a magnification of ×100 were calculated for each animal (*n* = 5 mice per group). **e** The mRNA expression of *p21*^*CIP1*^, *p27*^*KIP1*^, and *p15*^*INK4b*^ in the renal cortices from the four groups. The results are presented as the means ± SEMs. *P < 0.05, **P < 0.01, and ***P < 0.001, NS no significance between the indicated groups, and ^###^*P* < 0.001 compared with the other three groups, as analyzed by Tukey’s test after one-way ANOVA (*n* = 6–8 for each group)

We further determined the activity level of SA-β-gal, the most commonly used marker of cellular senescence, in the kidneys and found that similar to the accumulation of p16^*INK4a*^, positive staining in KO-Aging glomeruli increased significantly compared with that in WT-Aging glomeruli (Fig. 4a, b), which confirmed that *C/EBPα* deletion accelerated podocyte senescence upon aging. Moreover, SA-β-gal activity was significantly increased in aging cortices compared with those in young cortices; however, it was not further increased in the KO-Aging group compared with the WT-Aging group (Fig. 4c, d). In addition, we quantified the mRNA levels of cellular senescence markers, including the cyclin-dependent kinase inhibitors *p21*^*CIP1*^*, p27*^*KIP1*^, and *p15*^*INK4b*^ in the kidney cortices and found that they were increased significantly in the aging groups compared with the young groups (Fig. 4e). Consistent with the results of SA-β-gal staining, mice with podocyte-specific *C/EBPα* deletion exhibited no changes in the mRNA levels of these markers compared with those exhibited by wild-type mice of the same age (Fig. 4e). On the basis of our data, we hypothesized that C/EBPα knockout aggravates aging-induced podocyte senescence, which manifests as the impaired podocyte structure and function and the accumulation of senescent markers, but this accumulation is not significantly aggravated in the tubulointerstitium.

### Deletion of *C/EBPα* in podocytes exacerbates aging-induced glomerulosclerosis and tubulointerstitial EMT

**Fig. 5:**
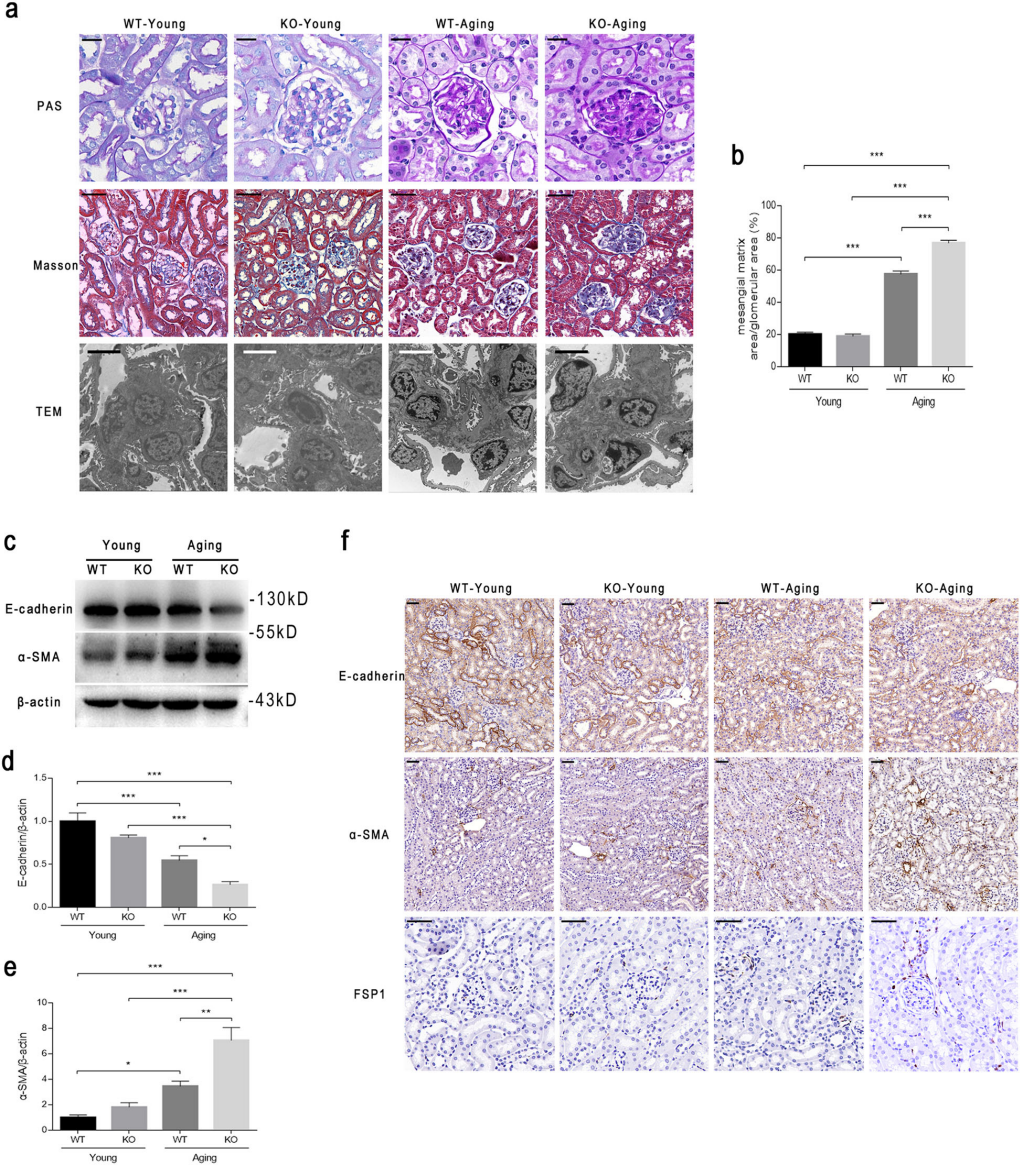
Podocyte-specific *C/EBPα* knockout aggravates aging-induced renal lesions. **a** Representative light micrographs of periodic acid-Schiff (PAS)-stained (row 1, scale bars = 20 μm), and Trichrome Masson-stained (row 2, scale bars = 50 μm) kidney sections and TEM micrographs (row 3, scale bars = 5.0 μm) from WT-Young, KO-Young, WT-Aging, and KO-Aging mice. **b** The quantification of the mesangial area fraction in the glomerular tuft from 10 to 15 randomly selected glomeruli per animal in each indicated group (*n* = 5 mice per group). **c** A comparison of the expression of EMT markers in the renal cortices of the four groups. β-actin served as a loading control. Densitometric analysis indicated a significant reduction in the expression of E-cadherin (**d**), and an increase in the expression of α-SMA (**e**) in aging *C/EBPα* knockout mice. **f** Representative immunohistochemical micrographs of E-cadherin (row 1), α-SMA (row 2), and FSP-1 (row 3) expression in the four groups described in (**e**). The scale bars = 50 μm. The results are presented as the means ± SEMs. *P < 0.05, **P < 0.01, and ***P < 0.001 between the indicated groups, as analyzed by Tukey’s test after one-way ANOVA (*n* = 6–8 for each group)

To further investigate whether C/EBPα loss in podocytes affects kidney injury caused by aging, we performed histological staining and TEM micrograph analysis and found that aging induced significant mesangial expansion and more substantial mesangial matrix accumulation in KO-Aging glomeruli than in WT-Aging glomeruli as measured by PAS staining (Fig. 5a, row 1 and Fig. 5b). Similarly, with Trichrome Masson staining and TEM, we found that the increase in the extracellular matrix in KO-Aging glomeruli was more serious than that in WT-Aging glomeruli (Fig. 5a, rows 2 and 3). In addition, the PAS-staining micrographs showed moderate tubular basement thickening in the WT-Aging group, but serious thickening in the KO-Aging group (Fig. 5a, row 1), and Trichrome Masson staining also showed increased tubulointerstitial fibrosis in the KO-Aging group compared with the other three groups (Fig. 5a, row 2). These results demonstrated that podocyte-specific *C/EBPα* knockout not only aggravates glomerulosclerosis in the setting of chronologic aging, but also induces the development of tubulointerstitial injury.

Our observations suggested greater tubular basement thickening in the KO-Aging group than in the WT-Aging group based on PAS staining despite similar degrees of tubular cell senescence based on SA-β-gal staining. To confirm that *C/EBPα* loss in podocytes aggravates tubulointerstitial epithelial–mesenchymal transdifferentiation (EMT), we isolated renal cortices and evaluated E-cadherin and α-SMA levels by western blotting. The expression levels of both E-cadherin and α-SMA showed no significant change between WT-Young and KO-Young mice; however, the level of E-cadherin decreased and the level of α-SMA increased significantly upon aging (Fig. 5c–e). The changes were more significant between the WT-Aging and KO-Aging groups (Fig. 5c–e). Then, we conducted immunohistochemistry (IHC) staining to determine the localization of EMT indicators. E-cadherin was mainly expressed in tubular cells, and α-SMA was expressed in the interstitium. The variations among the four groups were the same as those shown by western blot (Fig. 5f, rows 1 and 2). The positive area of FSP-1 in IHC staining, which was mainly located in the tubulointerstitium, also increased upon aging, and *C/EBPα* deletion in podocytes induced a greater accumulation of FSP-1 (Fig. 5f, row 3). Based on these findings, we concluded that the targeted knockout of *C/EBPα* expression in podocytes results in the exacerbation of glomerulosclerosis and tubulointerstitial EMT upon aging.

### Albuminuria in podocyte-specific *C/EBPα*-null aging mice aggravates tubulointerstitial EMT

**Fig. 6:**
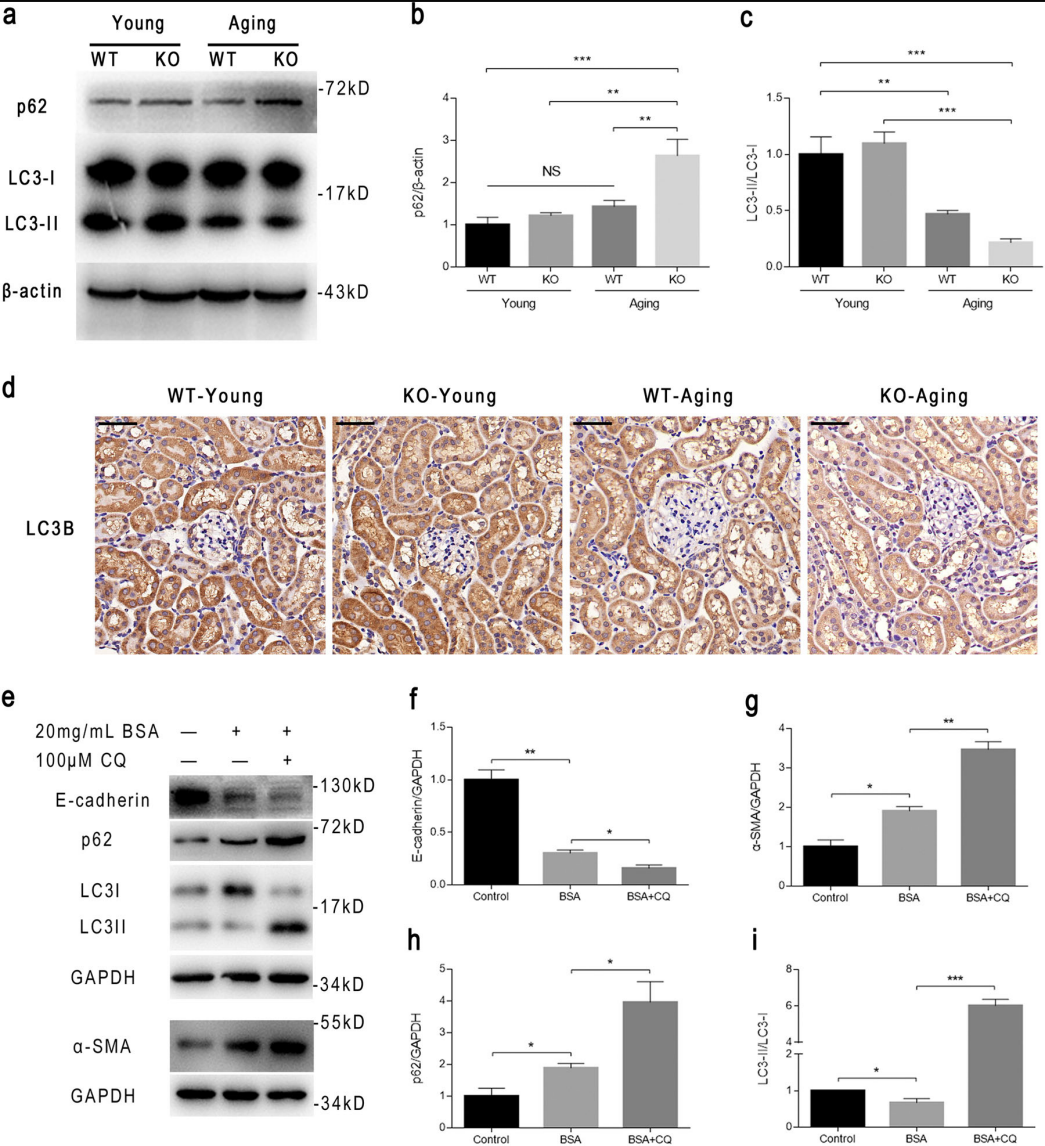
C/EBPα exerts effects on the autophagic activity of aging tubules in vivo and in vitro. **a** A comparison of the expression of the autophagic markers p62 and LC3-II/LC3-I in the renal cortices of the four indicated groups, as determined by western blotting. β-actin served as a loading control for p62. **b** The densitometric analysis of the bands in (**a**) indicating significantly increased p62 expression in KO-Aging mice. **c** The densitometric analysis of the bands in (**a**) showing significantly reduced LC3-II/LC3-I expression in the WT-Aging group compared with the WT-Young group and a more dramatic decrease in the KO-Aging group. **d** Representative immunohistochemical micrographs of LC3B in the four groups confirmed the altered expression observed by western blotting. **e** Western blot bands for EMT markers (E-cadherin and α-SMA) and autophagic markers (p62 and LC3-II/LC3-I) in HK-2 cells stimulated with 100 μM CQ + 20 mg/mL BSA for 24 h (the cell was prestimulated with CQ for 2 h before the addition of BSA). GAPDH served as a loading control. **f**–**i** The densitometric analysis of the bands in (**e**). The relative intensities in the WT-Young group were set to 1. The results are presented as the means ± SEMs. *P < 0.05, **P < 0.01, and ***P < 0.001, NS no significance between the indicated groups, as analyzed by Tukey’s test after one-way ANOVA (*n* = 6–8 for each group for the mouse experiments and *n* = 3 in triplicate for the cell experiments)

We found that podocyte-targeted *C/EBPα* deletion aggravates tubulointerstitial EMT in aging mice, but how genetic deletion in podocytes affects tubular phenotypes remains an intriguing question. It has been proposed that tubular cells exhibit a high level of constitutive autophagy, and autophagy deficiency accelerates tubular injury^9^. Therefore, we also investigated whether aging-induced EMT in tubular cells is accompanied by abnormal autophagy homeostasis. First, we determined the change in the expression of the autophagy markers p62 and LC3B in renal cortices by western blotting and found that p62 was upregulated slightly in WT-Aging mice compared with mice from both young groups, but aging reduced the expression of LC3-II significantly (by ~50%, *P* < 0.01) (Fig. 6a–c). Moreover, in the KO-Aging group, the expression level of p62 was significantly higher than that in all other groups, and LC3-II decreased more significantly (by ~80%, *P* < 0.001 as compared with the WT-Young group) than in the WT-Aging group (Fig. 6a–c). IHC staining of LC3B showed that it was mainly expressed in tubular cells, and the decrease in the positive area and degree indicated decreased autophagic activity in senescent tubules after the disruption of podocyte-specific C/EBPα expression (Fig. 6d). These findings indicate that dysregulated autophagy may be involved in the occurrence and progression of tubular EMT in a chronologic aging model.

We next employed HK-2 cells to construct the model and confirm the mechanism in vitro. Given that albuminuria was increased in the KO-Aging group compared with the WT-Aging group, HK-2 cells were stimulated by bovine serum albumin (BSA), and the western blotting results showed that stimulation with 20 mg/mL BSA for 24 h caused tubular cell EMT, which was demonstrated by a significantly reduced expression of E-cadherin, increased expression of α-SMA (Fig. 6e–g), and the suppression of autophagy, which was demonstrated by significantly increased levels of p62 and decreased levels of LC3-II/I (Fig. 6e, h, i). These results were consistent with the changes in tubular cells in WT-Aging mice in vivo. Adding 100 μM CQ (an autophagy inhibitor) in addition to 20 mg/mL BSA (BSA + CQ group) caused more dramatic increases in the levels of p62 and LC3-II/I (Fig. 6e, h, i), indicating that CQ blocked autophagosome fusion and degradation. A further reduction in E-cadherin expression and an increase in α-SMA expression (Fig. 5e–g) were observed in the BSA + CQ group compared with the BSA group. Taken together, these results suggest that albuminuria accelerates tubular EMT by blocking autophagy in aging kidneys.

### C/EBPα overexpression alleviates ADR-induced premature podocyte senescence

**Fig. 7:**
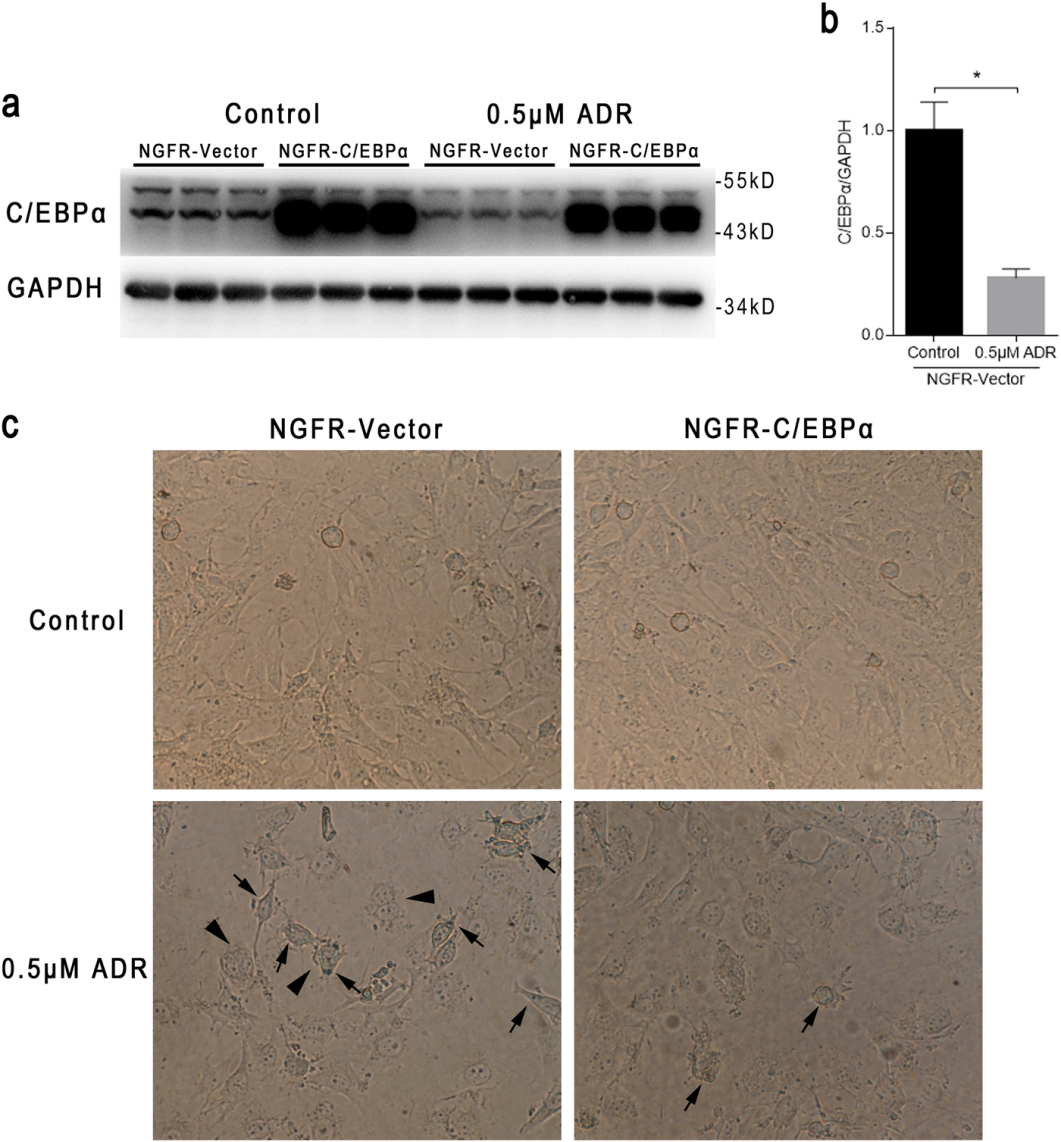
C/EBPα mitigates ADR-induced premature senescence in cultured podocytes. **a** Western blot analysis of C/EBPα in immortalized mouse podocytes stimulated with or without 0.5 μM ADR for 24 h after transfection with NGFR-C/EBPα or NGFR-vector plasmid for 24 h. **b** Semiquantitative average band intensity in (**a**). GAPDH served as a loading control. The results are presented as the means ± SEMs. *P < 0.05 between the indicated two groups, as analyzed by Student’s *t* test (*n* = 3 for each group). **c** Representative light micrographs of immortalized mouse podocytes exposed to the conditions indicated in (**a**), and then undergoing SA-β-gal staining. Magnification = ×400. The increased activity of SA-β-gal is indicated by arrows, and binucleate/multinucleate podocytes are indicated by triangles

We subsequently explored the role and mechanism of C/EBPα in podocyte senescence in vitro. ADR, a chemotherapeutic agent that causes DNA damage, was employed at a concentration of 0.5 μM to induce the premature senescence^10,11^ of podocytes, and we found that the expression level of C/EBPα decreased significantly in podocytes treated with ADR (0.5 μM ADR group) compared with podocytes not treated with ADR (control group) (Fig. 7a, b). Next, we investigated whether overexpressing C/EBPα can reverse ADR-induced senescence. The overexpression of C/EBPα was confirmed through western blotting (Fig. 7a), and the morphology and positive staining of SA-β-gal staining was not obviously altered by C/EBPα overexpression (Fig. 7c, upper row). However, ADR stimulation apparently aggravated senescent phenotypes (Fig. 7c, lower left corner), including enlarged podocytes, increased the activity of SA-β-gal (Fig. 7c, indicated by arrows) and binucleate/multinucleate podocytes (Fig. 7c, indicated by triangles), and C/EBPα overexpression substantially attenuated these morphologic characteristics of senescence and SA-β-gal activity accumulation (Fig. 7c, lower right corner).

**Fig. 8:**
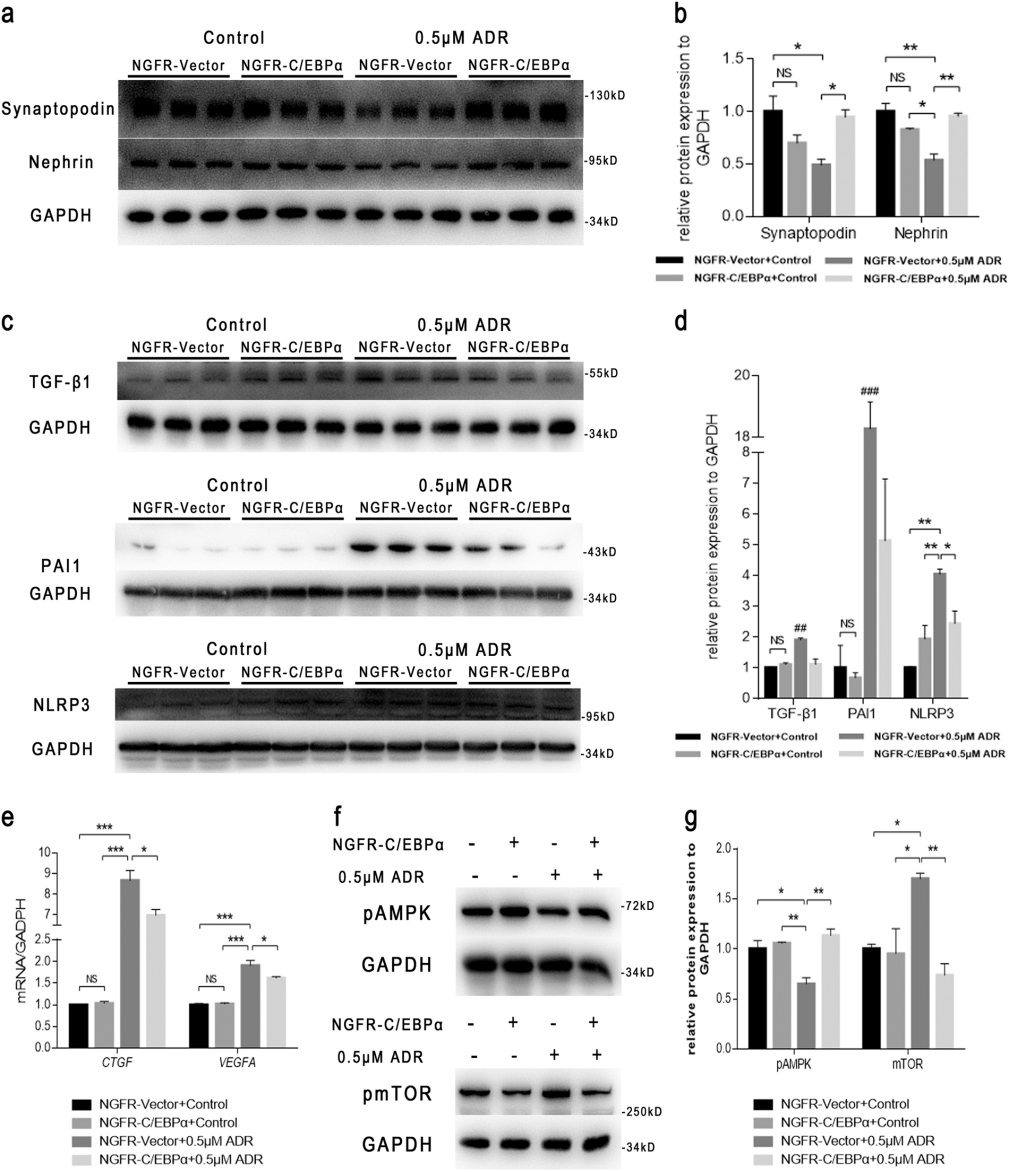
C/EBPα protects cultured podocytes from SASP in premature senescence. **a** Western blot analysis of synaptopodin and nephrin in immortalized mouse podocytes stimulated with or without 0.5 μM ADR for 24 h after transfection with the NGFR-C/EBPα or NGFR-vector plasmid for 24 h. **b** Semiquantitative average band intensity in (**a**). **c** Western blot analysis of TGF-β1, PAI-1, and NLRP3 in immortalized mouse podocytes that underwent the conditions indicated in (**a**). **d** Semiquantitative average band intensity in (**c**). **e** The mRNA expression of *CTGF* and *VEGFA* in the four indicated groups. **f** Western blot analysis of phospho-AMPK and phospho-mTOR in immortalized mouse podocytes exposed to the conditions indicated. **g** Semiquantitative average band intensity in (**f**). The results are presented as the means ± SEMs. GAPDH served as a loading control, and the value in the NGFR vector + control group was set to 1. *P < 0.05, **P < 0.01, and ***P < 0.001 between the indicated groups, as analyzed by Tukey’s test after one-way ANOVA (*n* = 3 in triplicate)

To test whether C/EBPα overexpression exerts a protective effect against ADR-induced podocyte senescence, we determined the protein levels of the podocyte markers synaptopodin and nephrin through western blotting, and found that the expression of both of these markers decreased significantly in induced senescent podocytes (NGFR vector + ADR) compared with control podocytes (NGFR vector + control), and C/EBPα overexpression reversed the decreased expression of synaptopodin and nephrin (NGFR-C/EBPα + ADR) (Fig. 8a, b). ADR stimulation also significantly induced the expression of TGF-β1, PAI-1, and NLRP3 (Fig. 8c, d), which demonstrates the senescence-associated secretory phenotype (SASP) of podocytes^12^; however, the expression of these proteins was significantly decreased in the NGFR-C/EBPα + ADR group compared with the NGFR vector + ADR group (Fig. 8c, d). Moreover, ADR stimulation induced the mRNA expression of *CTGF* and *VEGFA* in podocytes, and this effect was reversed by C/EBPα overexpression (Fig. 8e). In addition, C/EBPα overexpression reversed the reduction in phospho-AMPK expression and the increase in phospho-mTOR expression induced by ADR (Fig. 8f, g), which might suggest a potential molecular mechanism.

## Discussion

This study identified the importance of C/EBPα in podocyte senescence and its effect on aging-induced kidney injury. Although two previous studies reported the underlying relationship between C/EBPα and cellular senescence^13,14^, to our knowledge, this is the first study to elucidate the importance of C/EBPα in aging-related kidney injury. We found that the podocyte-specific deletion of *C/EBPα* accelerated podocyte senescence in vivo, which manifested as the accumulation of senescent markers in podocytes, unstable structure, impaired function, the synthesis of more extracellular matrix, and an increase in albuminuria. In addition, albuminuria aggravated tubulointerstitial EMT by inhibiting autophagy. Moreover, we confirmed the protective role of C/EBPα in ADR-induced premature senescence in cultured mouse podocytes.

Cell senescence has been proposed to be a state of irreversible growth arrest despite the maintenance of metabolic activity^15^. This does not mean that all cell types that permanently withdraw from the cell cycle, such as postmitotic neuron-like podocytes, which undergo terminal differentiation and exhibit functional and morphological changes during the process of differentiation and maturity, are senescent. As reported in the literature^16^, senescence in vitro can be divided into two types, namely, replicative senescence and induced senescence, which are the results of DNA damage with and without telomere shortening, respectively. The former is caused by mitosis and the latter is caused by exogenous stressors. Podocytes have no proliferative capacity in adult mammals or under completely differentiated conditions in vitro. Because these cells exit replication and the quiescent cell cycle means, it is difficult to model replicative or induced senescence based on completely differentiated podocytes in vitro. Therefore, we employed partially differentiated podocytes, which, like podocytes in infantile mammals, maintain some proliferative capacity and express synaptopodin, one of the most sensitive and specific markers of differentiated podocytes identified thus far^17^, to induce premature senescence by ADR.

We determined that podocyte-specific *C/EBPα* deletion aggravated podocyte chronologic senescence in vivo, and the genetic upregulation of *C/EBPα* improved ADR-induced premature senescent phenotypes, including mitotic catastrophe (MC) and SASP in vitro. Podocytes require an actin cytoskeleton to maintain their sophisticated morphological structure; however, binucleate/multinucleate podocytes lose their normal markers and foot processes. Cultured podocytes exposed to ADR exited mitosis, became binucleate/multinucleate, and underwent senescence. Aneuploid podocytes are a feature of MC both in vitro and in vivo, which indicates that they may detach and die over a short period of time^18^. SASP is a common characteristic of all types of senescence, and these alterations include the production of some secreted factors that enhance the senescence of the cell per se or the surrounding cells, such as plasminogen activator inhibitor-1 (PAI-1), its upstream TGF-β1^12^, and the NLRP3 inflammasome^19^, through paracrine and autocrine mechanisms^20^. Moreover, the upregulation of podocyte connective tissue growth factor (CTGF) and vascular endothelial growth factor (VEGF)-A has paracrine effects on mesangial cells that caused them to overproduce matrix proteins and ultimately contribute to mesangial matrix accumulation and glomerulosclerosis in chronic glomerular disease^18^. Subsequently, we tested the molecular mechanism of this process. AMP-activated protein kinase (AMPK) is a crucial sensor of energy, and a mammalian target of rapamycin (mTOR) has been identified as a senescence-regulated factor. The phosphorylation of the catalytic subunit AMPKα1 is known to phosphorylate the mTOR-binding partner Raptor directly, and Raptor phosphorylation inhibits mTOR activity^21^. mTOR complexes are one of the most conserved factors involved in aging, and genetic polymorphisms associated with reduced mTOR activity have been linked to longevity both in humans and in model organisms^22–24^. Researchers have suggested that mTOR promotes renal aging in vivo^25^ and renal tubular epithelial cell senescence in vitro^26^, but there have been few reports on the effects of mTOR on podocyte senescence. AMPK shuts down the mTOR complex to participate in energy-dependent senescence regulation and was demonstrated in this study to be a part of the molecular mechanism by which C/EBPα regulates podocyte senescence.

It has been reported that C/EBPα, in addition to playing a role in transcriptional regulation, influences cell fate by controlling proliferation, including the induction and stabilization of p21^*CIP1*^ ^27^, and the direct interaction and inhibition of kinases cdk2 and cdk4^28^, and E2F-Rb complexes^29^. These molecules are also important markers of the p53-Rb senescent pathway. Our experiments on cultured podocytes showed that the expression of these markers was induced by ADR, but C/EBPα overexpression had no effect on the levels of these markers (data not provided). In addition, we found that under aging conditions, both protein and mRNA levels of podocyte markers, nephrin, podocin, synaptopodin, and WT-1, varied significantly with knockout/overexpression of C/EBPα in vivo and in vitro; however, the effects of C/EBPα on the four podocyte markers were mild and with no significance in protein and mRNA levels in the young mice. These findings suggest that the effects of C/EBPα on the four markers are senescence-dependent and through different molecular mechanisms under young and aging conditions. Given that C/EBPα promotes metabolism and inhibits inflammation in macrophages^30,31^ and that metabolic and inflammatory disorders are important mechanisms for promoting cell senescence, we hypothesized that C/EBPα inhibits podocyte senescence by regulating metabolism and inflammation, and we subsequently confirmed this scientific hypothesis. We revealed that C/EBPα regulates podocyte senescence through mechanisms associated with metabolism (the AMPK/mTOR pathway in this study) or inflammation (the NLRP3 inflammasome in this study), but not with cell cycle proteins or the p53-Rb pathway. However, it remains unclear which genes are directly transcriptionally regulated by C/EBPα, as a transcriptional factor, to mitigate podocyte senescence.

C57BL/6 mice might be an appropriate model for studying kidney aging because they, like humans, do not develop proteinuria upon physiological aging, but instead show a loss of functional nephrons as the main phenotype^32^. As terminally differentiated cells with minimal proliferation capacity, podocytes play a central role in renal aging^33^. Recent observations have suggested that podocyte depletion causes glomerulosclerosis in various glomerulopathies, such as diabetic glomerulosclerosis, IgA nephropathy, and hypertensive glomerulosclerosis^34^, followed by subsequent tubular injury and interstitial fibrosis^35^. After C/EBPα was specifically knocked out in podocytes in our study, significant albuminuria, which was accompanied by greater podocyte senescence and depletion occurred in aging mice. Therefore, we consider podocyte senescence to be the initiating process of aging-related glomerulosclerosis and tubular injury. Subsequently, we verified one of the mechanisms that podocyte senescence leads to tubular injury by employing the cultured HK-2 cells in vitro; albumin overload was one of the causes and autophagy inhibition was its mechanism. Tubular cells are epithelial cells with high-energy consumption and high levels of constitutive autophagy and autophagy diminishes with aging^32^. Autophagy repression enhances tubular cell apoptosis and interstitial fibrosis^8^. Therefore, we focused mainly on autophagy and found that autophagy activity was decreased with increased tubular EMT in both aging mice and BSA-stimulated HK-2 cells, and the induction of EMT by BSA was enhanced by the inhibition of autophagy by CQ in vitro.

In summary, our findings identified C/EBPα in podocytes as a crucial factor for podocyte senescence and kidney homeostasis upon aging, and the AMPK/mTOR pathway as the molecular mechanism. In addition, we demonstrated that aging-related tubular EMT was aggravated by albuminuria and reduced autophagic activity. The findings reported herein may be pertinent not only to chronologically aging animals, but also premature senescence, and these insights may offer new approaches for the treatment and prevention of the course of renal injury in aging.

## Supplementary information

Supplementary Table 1. Antibody validation profile.

Supplementary Figure 1. Original scans of immunoblots.
