# Supplementary material for: C/EBPα deficiency in podocytes aggravates podocyte senescence and kidney injury in aging mice
Source: Cell Death Dis. 2019 Sep 17;10(10):684. doi: 10.1038/s41419-019-1933-2 (PMC6746733; doi:10.1038/s41419-019-1933-2)
Supplement: Supplementary file 1 — Supplementary Table 1. Antibody validation profile. [file 41419_2019_1933_MOESM1_ESM.pdf]

Supplementary Table 1. Antibody validation profile

**Primary Antibody**	**Clone**	**Campany**	**Catalog No.**	**Dilution**
C/EBPα	Polyclonal	abcam	ab140479	1:1000 (WB)
C/EBPα	Monoclonal (D-5)	Santa Cruz	sc-365318	1:100 (IF)
Synaptopodin	Polyclonal	Santa Cruz	sc-21537	1:500 (WB)<br>1:100 (IF)
Nephrin	Polyclonal	abcam	ab58968	1:300
CDKN2A/p16INK4a	Monoclonal (2D9A12)	abcam	ab54210	1:500 (IF)
WT1	Monoclonal (F-6)	Santa Cruz	sc-7385	1:200 (IF)
E-cadherin	Monoclonal (M168)	abcam	ab76055	1:1000 (WB)<br>1:200 (IHC)
α-SMA	Monoclonal (CGA7)	Santa Cruz	sc-53015	1:500 (WB)<br>1:100 (IHC)
S100A4	Polyclonal	abcam	ab41532	1:100 (IHC)
SQSTM1/p62	Polyclonal	Cell Signaling	5114	1:1000
LC3B	Polyclonal	Cell Signaling	2775	1:1000 (WB)<br>1:200 (IHC)
TGFβ1	Polyclonal	ABclonal	A2124	1:1000
PAI1	Polyclonal	abcam	ab66705	1:1000
NLRP3	Monoclonal (Cryo-2)	AdipoGen	AG-20B-0014	1:1000
Phospho-AMPKα(Thr172)	Monoclonal (40H9)	Cell Signaling	2535	1:1000
Phospho-mTOR (Ser2448)	Monoclonal (D9C2)	Cell Signaling	5536	1:1000
GAPDH	Monoclonal (6C5)	abcam	ab8245	1:5000
β-actin	Monoclonal (AC-15)	Sigma	A5441	1:5000
**Secondary antibody**	**Conjugate(s) Used**	**Campany**	**Catalog No.**	**Dilution**
Donkey anti-Goat	HRP	Santa Cruz	Sc-2020	1:5000 (WB)
Anti-mouse IgG	HRP	Cell Signalling	7076	1:2000 (WB)
Anti-rabbit IgG	HRP	Cell Signalling	7074	1:2000 (WB)
Donkey anti-Mouse	Alexa Fluor® 594	Invitrogen	A21203	1:500 (IF)
Donkey anti-Mouse	Alexa Fluor® 488	Invitrogen	A21202	1:2000 (IF)
Donkey anti-Goat	Alexa Fluor® 594	Invitrogen	A11058	1:200 (IF)
Goat anti-Rabbit	HRP	ZSGB-BIO (China)	PV-6001	Undiluted (IHC)
Goat anti-Mouse	HRP	ZSGB-BIO (China)	PV-6002	Undiluted (IHC)
