# Supplementary figures and images for: C/EBPα deficiency in podocytes aggravates podocyte senescence and kidney injury in aging mice
Source: Cell Death Dis. 2019 Sep 17;10(10):684. doi: 10.1038/s41419-019-1933-2 (PMC6746733; doi:10.1038/s41419-019-1933-2)

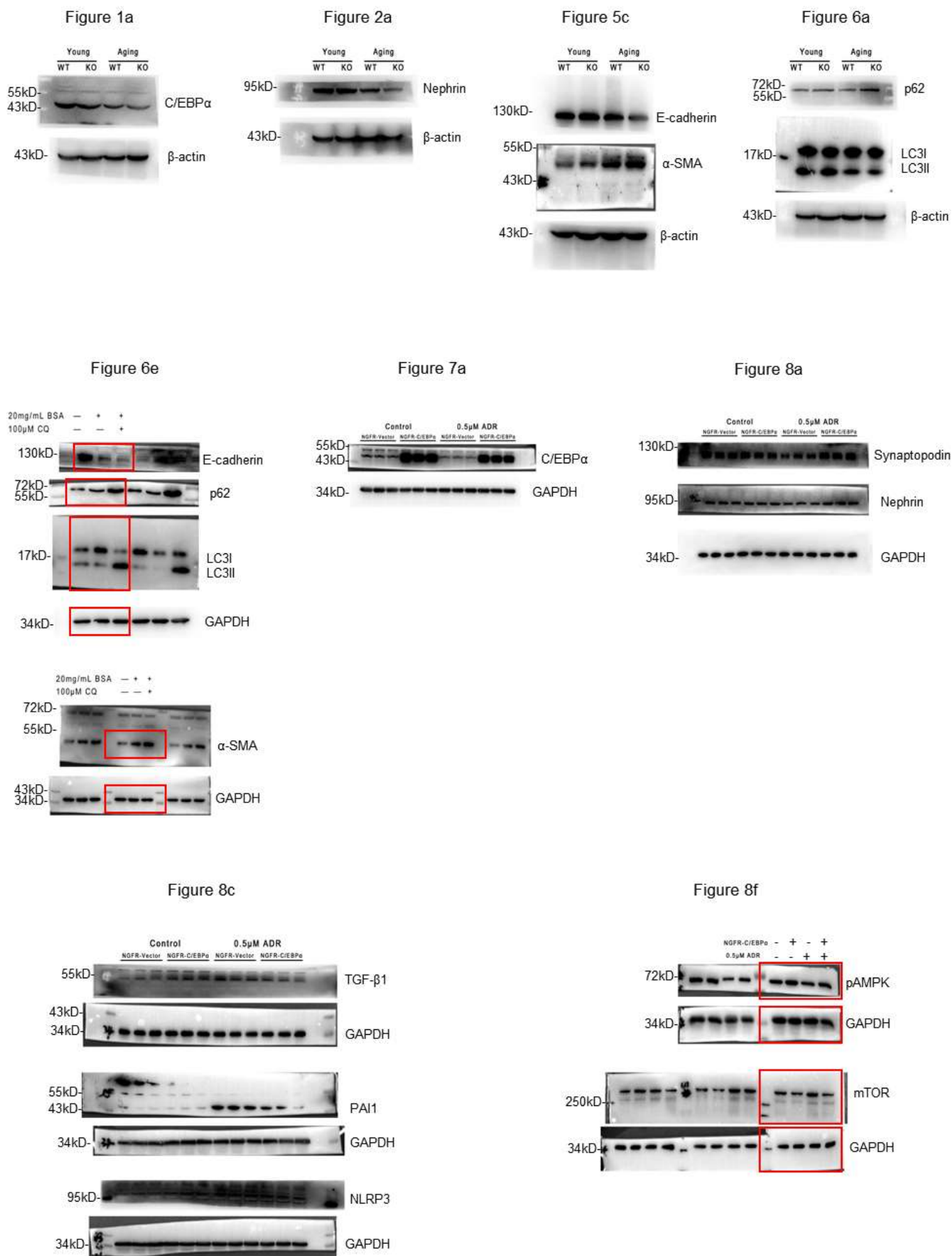

**Supplementary Figure 1. Original scans of immunoblots**

Supplement: Supplementary file 2 — Supplementary Figure 1. Original scans of immunoblots. [file 41419_2019_1933_MOESM2_ESM.pdf]
